# Cell culture and genetic transfection methods for the Japanese scallop, *Patinopecten yessoensis*

**DOI:** 10.1002/2211-5463.13237

**Published:** 2021-07-16

**Authors:** Minako Suzuki, Tomomi Okumura, Koki Uchida, Yukinori Ikeda, Yasuhiro Tomooka, Tadaaki Nakajima

**Affiliations:** ^1^ Department of Biological Science and Technology Faculty of Industrial Science and Technology Tokyo University of Science Katsushika‐ku Japan; ^2^ Department of Basic Biology School of Life Science The Graduate University for Advanced Studies SOKENDAI Okazaki Aichi Japan; ^3^ Institute of Industrial Science The University of Tokyo Meguro‐ku Japan; ^4^ Department of Science Yokohama City University Kanazawa‐ku Japan

**Keywords:** genetic transfection, gonadal cells, primary culture, scallop

## Abstract

Cell cultures can simplify assays of biological phenomena; therefore, cell culture systems have been established for many species, even invertebrates. However, there are few primary culture systems from marine invertebrates that can be maintained long term. The Japanese scallop, *Patinopecten yessoensis*, is a marine bivalve. Cell culture systems for the scallop have only been established for a few organ‐derived cell types and for embryonic cells. We developed a primary culture system for cells from male and female scallop gonads, hepatopancreas, and adductor muscle by utilizing culture conditions closer to those in nature, with regard to temperature, osmolarity, and nutrition. Primary cultured female gonadal cells were maintained for more than 1 month and had potential for proliferation. Furthermore, a genetic transfection system was attempted using a scallop‐derived promoter and a lipofection reagent. GFP‐positive cells were detected in the attempt. These technical developments would promote our understanding of biochemical mechanisms in scallops as well as providing clues for establishment of immortalized molluscan cell lines.

## Graphical abstract

We developed a primary culture system for female and male gonad‐, hepatopancreas‐, and adductor muscle‐derived cells from Japanese scallop, *Patinopecten yessoensis*. In the female gonadal cell culture, we succeeded in maintaining the cultured cells for at least one month and the culture had potential for cell proliferation. We attempted transfection of female gonadal cells with scallop‐derived promoters and lipofection reagent.

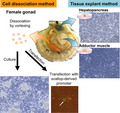

**Abbreviations**
AF: autofluorescence
AME: adductor muscle extract
BLC: bovine lipoprotein‐cholesterol
BSA: bovine serum albumin
MF: multiple filopodia
PE: phosphoethanolamine
TP: tryptose phosphate broth

As cell culture can simplify assays of biological phenomena, cell culture systems have been established for many species. Normally, cells from primary culture arrest cell proliferation and eventually die in long‐term culture [1]. Immortalized cell lines are established either by a spontaneous mutation in culture or by genetic manipulation of oncogenes or genes involved in cell proliferation in a stable primary culture system [1]. Immortalized cell lines have been established in many species, including invertebrates. There are hundreds of cell lines from insects [2]. However, there is only one immortalized cell line from a mollusk, the Bge cell line derived from a snail, *Biomphalaria glabrata* [3]. Furthermore, among marine invertebrates, immortalized cell lines have been established only for *Acropora tenuis* [4]. Although culture conditions from other studies may apply, cell culture techniques are not as advanced for mollusks and other marine invertebrates, as they are for model organisms [5, 6].

Many taxa in the Phylum Mollusca are marine [7]. In most cell culture studies of marine mollusks, hemocytes have been used; however, there are few reports regarding somatic cell cultures [8, 9]. In the Pacific oyster, *Crassostrea gigas*, a primary culture system was developed with a variety of morphological cell types from large tissue explants [9]. Gonadal cell cultures were developed for this species and for the hard clam, *Meretrir lusoria*, with some morphological cell types from tissue explants [9, 10]. Cell cultures of digestive gland‐derived amoebocytes, adductor muscle‐derived spindle‐shaped cells, and cells with elongated protrusions were also developed from Pacific oyster tissue explants [9, 10]. However, there are few reports of long‐term culture and cell proliferation potential has not been analyzed in marine mollusk primary cell cultures [5, 8], suggesting limited proliferation potential. There is ample room to improve culture conditions by more closely approximating natural temperature, osmolarity, and nutrition.

The Japanese scallop, *Patinopecten yessoensis*, is a marine bivalve with a huge, edible adductor muscle and unusual biology, including sex reversal and accumulation of heavy metals in its hepatopancreas [11, 12]. Since scallops spawn once a year and the degree of maturation differs by season, establishment of a primary culture system and immortalized cell lines is important for structural and ecological analysis [13]. Without hemocytes, scallop cell culture was achieved with embryonic cells and mantle cells [13, 14]. The embryonic cell culture employed the cell dispersion method with collagenase, and cells were cultured on collagen‐coated dishes [13]. The mantle cell culture utilized the tissue explant method, and cells were cultured on noncoated dishes [14]. However, there are no reports of primary cultures from other organs.

We developed a primary culture system for scallop cells from male and female gonads, hepatopancreas, and adductor muscle. We attempted to bring culture conditions closer to those in nature by controlling temperature and osmolarity and by providing nutritional supplements. Primary cultured female gonadal cells could be maintained at least 1 month by modification of fundamental conditions. Furthermore, with additional supplements, primary cultured gonadal cells appeared to have potential for proliferation. Furthermore, GFP‐expressing cells were detectable with the transfection system developed using scallop‐derived promoters and a lipofection regent.

## Materials and methods

### Animals

Two‐year‐old scallops (*P. yessoensis*) cultured along the coast of Sanriku, Iwate, Japan (Yamakichi, Kamaishi, Japan), were used. Scallop sex was identified by observation of gonadal color. pH and osmolality in each organ were measured using a BioProfile 400 (Nova Biomedical, Waltham, MA, USA).

### Primary culture for female scallop gonadal cells

Female scallop gonads were dissected into pieces of ~ 3 mm^3^. Tissues were dissociated by vortexing with 4 pulses every 30 min in artificial seawater, SEALIFE (Nihon Kaisui, Tokyo, Japan), for 2 h at 10 ℃. Dissociated cells were filtered through a 70‐μm nylon mesh (FALCON, Franklin Lakes, NJ, USA) and washed 3 times with SEALIFE, including antibiotics (Table S1). Washed cells were centrifuged at 700 ***g*** for 10 min at 4 ℃. Dissociated cells were seeded on collagen‐, gelatin‐, poly‐D‐lysine‐, and scallop mantle extract‐coated dishes with basal medium [Leibovitz L‐15 medium (SAFC Biosciences, Lenexa, KS, USA), medium 199 (Sigma, St Louis, MO, USA), NCTC‐135 medium (Sigma), and Schneider’s insect medium (Sigma)] dissolved in SEALIFE (osmolality was adjusted to 960 or 1050 mosmol) with supplements (Table S2). These media are designated modified L‐15 (ML‐15), M199, MNCTC‐135, and MSchneider, respectively. Cells were cultured at 4, 10, or 15 ℃ with a half‐volume medium change every 3 days. For stable comparisons and to reduce variation among individual scallops, experimental groups in the same figure were derived from the same cell suspension, in which gonads of several scallops were mixed.

The additional supplements were added to ML‐15 (Table S3). Dishes were coated with 0.1% gelatin (Sigma), mouse type I collagen, and scallop mantle solution. For scallop mantle extract‐coated dishes, pieces of scallop mantle were homogenized in 25 mL SEALIFE. Homogenates were centrifuged at 1500 ***g*** for 10 min and supernatants were collected. Before culture, coated dishes were washed 3 times with SEALIFE. To prepare scallop adductor muscle extract (AME), an adductor muscle was frozen in liquid nitrogen, then crushed with a mortar, and diluted with 20 mL SEALIFE containing 1% acetic acid. The diluted sample was filtered overnight and centrifuged at 4 ℃ at 15 000 rpm. The supernatant was sterilized by passing it through a 0.45‐μm filter.

Uchida modified L‐15 (UML‐15) was made by adding 3 types of solution and bovine serum albumin (BSA) to ML‐15 (Table S4). To remove precipitate, the medium was centrifuged at 2000 rpm for 10 min and sterilized by passage through a 0.45‐μm filter. These media were stored at −30 ℃ until use.

### Passage of cultured female gonadal cells in UML‐15

Cultured cells were detached using a scraper. After centrifugation and aspiration of the supernatant, the pellet was suspended in UML‐15 and seeded into new dishes.

### Primary cultures of scallop hepatopancreas, adductor muscle, and male gonads

Scallop cells from hepatopancreas, adductor muscle, and male gonads were cultured using the tissue explant method. Each organ was dissected into pieces of ~ 5 mm^3^. Tissues were washed 8x with SEALIFE, including antibiotics, as mentioned above. After washing, tissues were explanted onto dishes for each type of condition (Table S5) and incubated at 10 ℃ for 3 days. For cell culture of adductor muscle, tissues were explanted on dishes to which the muscle fibers were attached vertically against the dishes. After incubation, tissues were removed and continuously cultured with a half‐volume medium change every 3 days.

### Cell Counting Kit‐8 (CCK‐8) assay for primary cultured scallop cells

Cell counting was performed using the CCK‐8 method (Dojindo, Kumamoto, Japan). Female gonadal cells were washed twice with SEALIFE. One mL of SEALIFE with 100 μL of CCK‐8 solution was added and then incubated for 1–4 h at room temperature. After incubation, absorbance at 450 nm was measured with a U‐2900 Spectrophotometer (Hitachi, Tokyo, Japan).

### Detection of cell proliferation

Proliferation of female gonadal cells was detected using a Click‐iT EdU Cell Proliferation Kit for Imaging, with Alexa Fluor 594 dye (Thermo Fisher Scientific, Waltham, MA USA). EdU solution was added to the culture medium and incubated for 22 h under culture conditions. After incubation, the medium with EdU solution was removed by aspiration, and cells adhered to dishes were fixed with 4% formaldehyde/PBS for 15 min at room temperature. After washing with 3% BSA/PBS, cells were permeabilized with 0.5% Triton X‐100/PBS for 20 min. Cells were treated with the kit reaction solution for 30 min at room temperature. For staining nuclei, 5 μg·mL^−1^ Hoechst solution was added to the samples.

### Isolation of housekeeping gene promoters and vector construction

*P. yessoensis* gDNA was extracted and purified from scallop gonads using the conventional phenol/chloroform extraction method. Genomic DNAs were extracted from tissues and cultured cells with SDS and proteinase K, purified using the conventional phenol/chloroform extraction method, precipitated with ethanol, and collected using wooden picks [15]. Predicted promoter regions of *P. yessoensis* housekeeping genes (*PyActb*, *PyGpx*, *PyHps70,* or *PyeIF3*) were obtained by genomic PCR with each primer (Table S6) using AmplitaqGold (Applied Biosystems, Weiterstadt, Germany). Promoters were inserted into pMD20‐T vectors (Promega, Madison, WI, USA) with ligation high ver.2 (TOYOBO, Osaka, Japan) and transfected into DH5α. After cloning, each promoter was subcloned into pEGFP‐N1 (Clontech Inc., Palo Alto, CA, USA).

### Transfection in female scallop gonadal cells

Female gonadal cells were transfected by 3 methods, electroporation, and lipofection using 2 types of solution, ScreenFect A (Fujifilm Wako, Osaka, Japan) and Lipofectamine 2000 (Thermo Fisher Scientific). For both solutions, lipofection was performed using the supplier’s protocol. For ScreenFect A, 2.5 μg DNA was added to 15 μL reagent in 125 μL of solution. For Lipofectamine, 4 μg DNA was added to 10 μL reagent in 250 μL of solution.

For electroporation, the suspension of female gonadal cells was added to electroporation buffer (130 mm NaCl, 5.3 mm KCl, 1.1 mm Na2HPO4, and 6.1 mm glucose) including 1 ng·μL^−1^ pEGFP‐N1 inserted with *PyActb*, *PyGpx*, *PyHps70,* or *PyeIF3*. Cells were subjected to electrical pulse [voltage (V):100, pulse length (ms): 0.05, number of pulses: 3, pulse interval (sec): 0.1] with transfer pulse [voltage (V): 25, pulse length (ms): 50, number of pulses: 10, pulse interval (sec): 0.1] by Gene Pulser Xcell (Bio‐Rad, Hercules, CA USA). Electroporated cells were immediately seeded onto dishes after electroporation.

### RT‐PCR analysis

RNA was isolated from hepatopancreas and adductor muscle. These organs were homogenized with a mix that consisted of 0.47 g·mL^−1^ guanidium thiocyanate, 7.3 mg·mL^−1^ sodium citrate dihydrate, 0.5% sodium N‐lauryl sarcosinate, and 2.5 mm dithiothreitol (Fujifilm Wako). mRNA was extracted using a Total RNA Extraction Column (FAVORGEN BIOTECH CORP., Ping‐Tung, Taiwan). Total RNA quality was assessed by agarose gel electrophoresis. cDNA was synthesized using 0.5 μg/10 μL of total RNA with ReverTra Ace Master Mix with gDNA Remover (TOYOBO). RT‐PCR was performed using AmpliTaqGold with primers in each condition (Table S7).

### Histological analysis

Histological analysis of hepatopancreas and adductor muscle was performed with HE staining. Tissues were fixed with Bouin's solution for 16 h and dehydrated with a graded alcohol series. Then, they were embedded in paraffin and sectioned at 8 μm.

### Statistical analyses

Data were summarized as means ± standard error. Two‐tailed Student’s *t*‐tests or Welch *t*‐tests were used for single comparisons. Tukey–Kramer or Games–Howell tests were used for multiple comparisons. A statistically significant difference was defined as *P* ≤ 0.05.

## Results

### Development of a fundamental primary culture system for female scallop gonadal cells

**Fig. 1:**
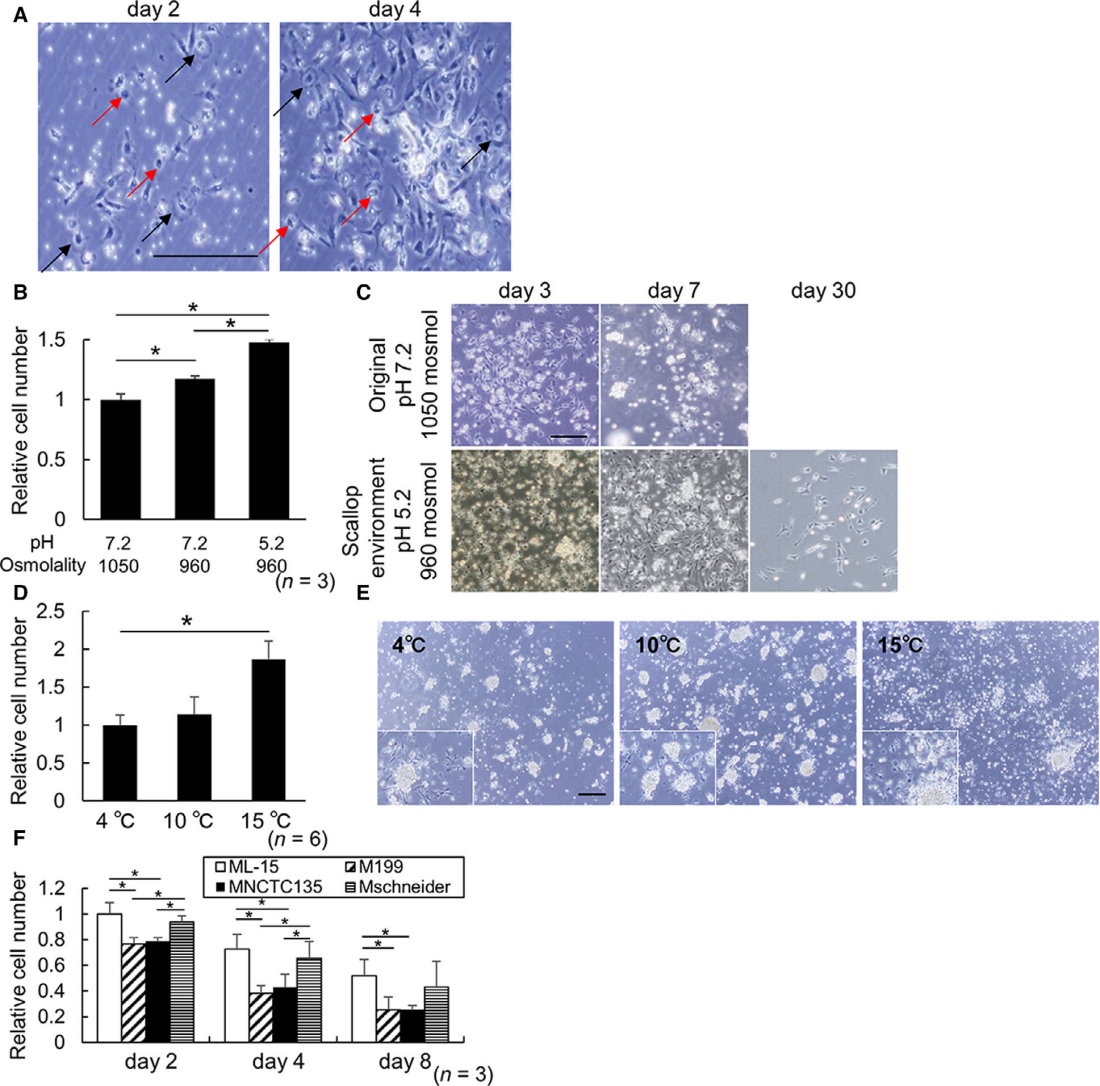
Development of a fundamental primary culture system for female scallop gonadal cells. (A) Phase‐contrast images of cells cultured on a gelatin‐coated dish at 2 and 4 days. Black arrows, spread adhered cells. Red arrows, round adhered cells. Scale bar: 200 μm. (B) Relative cell numbers analyzed by CCK‐8 in media under each pH and osmolality condition at 3 days. (pH 7.2 and 1050 mosmol; =1.0). Error bars: standard error, *P < 0.05, *n* = 3. (C) Phase‐contrast images of cultured cells in media under each pH and osmolality condition from 3 to 30 days. Scale bar: 200 μm. (D) Relative cell number analyzed by CCK‐8 at each temperature at 3 days. (4 ℃ = 1.0). Error bars: standard error, *P < 0.05, *n* = 6. (E) Phase‐contrast images of cultured cells at each temperature. Scale bar: 100 μm and 50 μm at higher magnification. (F) The relative number of cultured cells in each medium from 2 to 8 days. (ML‐15 at 2 days; =1.0). Error bars: standard error, *P < 0.05, *n* = 3.

We first examined the fundamental condition of primary culture for female gonadal cells. Because the efficiency of cell adhesion varied among individual scallops, we used cells from the same cell suspension to examine each condition. Female gonadal cells only adhered to gelatin‐coated dishes and cells were maintained for about 1 week (Figs 1A and S1A). Mainly fibroblast‐like cells and some round adhered cells and spread adhered cells were observed on days 2 and 4 (Fig. 1A). Cell numbers appeared to have increased by day 4 compared with day 2 (Fig. 1A). For quantitative comparison of culture conditions, a cell counting system was established using CCK‐8. CCK‐8 absorbance and seeded cell number were highly correlated (Fig. S1B).

For further improvement of the culture system, pH and osmolality were examined with L‐15 culture media and seawater (pH 7.2, 1050 mosmol) and native female scallop gonads (pH 5.2, 960 mosmol; Fig. 1B,C). Adaptation of pH and osmolality in the medium to the native female scallop gonadal environment significantly increased the cell number at day 2, as measured by CCK‐8 (Fig. 1B). Furthermore, this adaptation allowed culture for 30 days (Fig. 1C). We cultured female gonadal cells at 10℃ to examine coat treatment, pH, and osmolality. For further improvement, we tested incubation temperatures from 4 to 15 ℃ because mantle or embryonic cells in other scallop primary culture systems are cultured at 4 or 15 ℃, respectively [13, 14]. The number of cells cultured at 15 ℃ was significantly higher than at 4 ℃ (Fig. 1D). However, a smaller number of plural filopodia was detected among fibroblast‐like cells at 15 ℃ than in other conditions (Fig. 1E). Adherent cells tended to localize around the cell mass at all temperatures, and cell masses had a tendency to decrease and rapidly collapse at 15 ℃.

The basic media, Leibovitz L‐15, medium 199, NCTC‐135, and Schneider medium, commonly used for protostome cell culture, were employed with modifications of pH and osmolality (Fig. 1F) [16, 17]. The number of cells cultured in ML‐15 and MSchneider was higher than in M199 and MNCTC‐135 until day 8 (Fig. 1F). Cell numbers decreased in all media with time (Fig. 1F). Therefore, a fundamental primary culture system for female scallop gonadal cells was developed by comparing these conditions (dish‐coating, pH, osmolarity, temperature, and basal media).

### Improvement of a fundamental primary culture system for female scallop gonadal cells

**Fig. 2:**
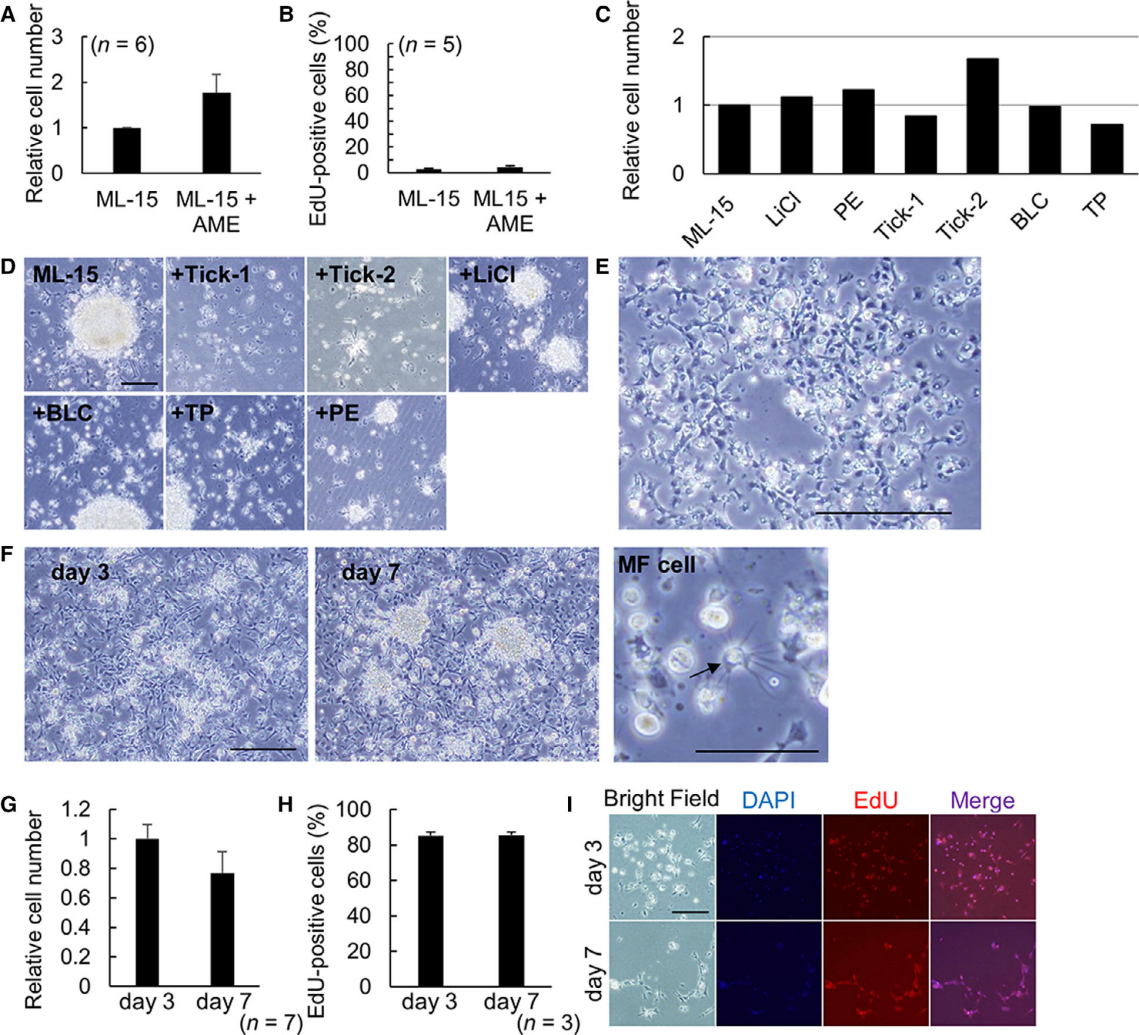
Improvement of a fundamental primary culture system for scallop gonadal cells. (A) The relative number of cultured cells in ML‐15 with or without AME at 3 days. (ML‐15; =1.0). Error bars: standard error, *n* = 6. (B) The ratio of EdU‐positive cells cultured in ML‐15 with or without AME at 3 days. Error bars: standard error, *n* = 5. (C) The relative number of cultured cells in ML‐15 with each supplement (ML‐15; =1.0). Each experiment comprised 2 or 3 replicates. (D) Phase‐contrast images of cultured cells with each supplement. Scale bar: 100 μm. (E) Phase‐contrast image of passaged cells, 3 days after passage. Scale bar: 100 μm. (F) Phase‐contrast images of cultured cells in UML‐15 for 3 and 7 days, and a high magnification image. Black arrow, MF with round cell body. Scale bar: 100 μm (G) The relative number of cultured cells in UML‐15 for 3 and 7 days. (3 days; =1.0). Error bars: standard error, *n* = 7. (H) The ratio of EdU‐positive cells to cultured cells with UML‐15 for 3 and 7 days. Error bars: standard error, n = 3. (I) Phase‐contrast, DAPI‐stained, and EdU‐incorporating images of cultured cells in UML‐15 for 3 and 7 days. Scale bar: 200 μm.

Because the fundamental culture system for female gonadal cells was not stable, we examined additional factors. However, factors commonly used in mammalian cell culture, such as FBS, did not affect scallop cell numbers (data not shown). Addition of 1% AME, which may include specific nutrients for scallops, increased the relative cell number and cell proliferation (Fig. 2A,B). However, even under these conditions, the cell proliferation rate remained low, although the incorporation time for EdU was 22 h (Fig. 2B). To increase cell proliferation, six more supplements were added to ML‐15, as well as AME for screening suitable supplements (Fig. 2C, Table S3). Cell numbers in media with LiCl, phosphoethanolamine (PE), and Tick‐2 tended to be higher than in ML‐15 (Fig. 2C). Cultured cells in each medium were mainly fibroblast‐like cells and a few round adhered cells (Fig. 2D). Because LiCl, PE, and Tick‐2 all tended to increase cell numbers individually, these supplements were added together in ML‐15 with AME (designated as UML‐15). The primary culture in UML‐15 was stable and could be passaged with scraping (Fig. 2E,F,H,I). Passaged cells tend to cluster in the dishes (Fig. 2E). Morphology of cells cultured in UML‐15 and passaged cells was largely similar to that in ML‐15, and cells having multiple filopodia (MF) with round cell bodies (MF cell) were newly detected in UML‐15 (Fig. 2F). While not statistically significant, the relative cell number tended to decrease with time (Fig. 2G). The cell proliferation rate in UML‐15 with EdU for 22 h reached 80% on days 3 and 7 and was drastically improved compared with only AME added to ML‐15 (Fig. 2B,H,I). Therefore, UML‐15 promoted stable and proliferative culture of female scallop gonadal cells.

### Attempt to establish a transfection system for scallop gonadal cells

**Fig. 3:**
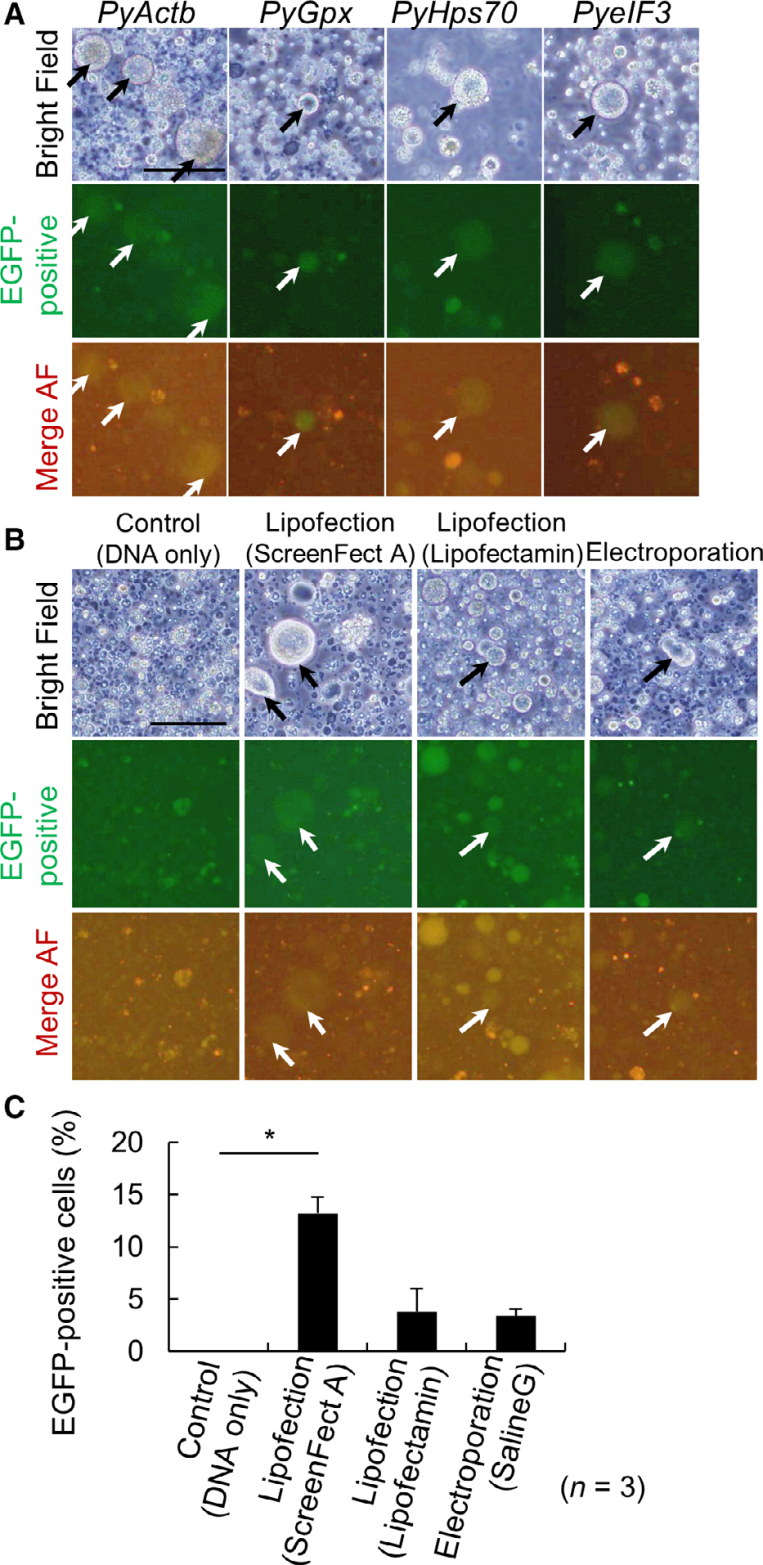
Attempt to establish a transfection system for scallop gonadal cells. (A) Phase‐contrast images of transfected cells with each promoter at 3 days. Black and white arrows, EGFP‐positive cells. Scale bar: 100 μm. AF: autofluorescence. (B) Phase‐contrast images of transfected cells by method at 3 days. Black and white arrows, EGFP‐positive cells. Scale bar: 100 μm. AF: autofluorescence. (C) The ratio of EGFP‐positive cells to cultured cells by method. Error bars: standard error, *P < 0.05. *n* = 3.

To establish a transfection system, active constructs in scallop cells were required. First, we attempted to obtain *P. yessoensis* promoter arrangements for *PyActb*, *PyGpx*, *PyHsp70,* and *PyeIF3*, which were expected to be active in all scallop cells. Each promoter activity was examined by observing EGFP fluorescence after transfection by lipofection using ScreenFectA (Fig. S2A‐Part A). Unfortunately, only weak EGFP intensity was detected in each experiment; however, we found that almost all the scallop female gonadal cells without transfection exhibited similar intensity of green and red autofluorescence (AF). Thus, for detection of GFP‐positive cells, we compared the green and red channels to exclude AF. EGFP‐positive cells were detected in all constructs at day 3, which suggested that all of four promoters were active in female gonadal cells (Fig. 3A). EGFP‐positive cells were large and round and did not appear to adhere to the dish (black and white arrows; Fig. 3A).

For improvement of the transfection system, the other three methods were examined again using *PyeIF3* (Fig. S2A‐Part B). Although EGFP intensity was low, EGFP‐positive cells were detected with all methods (Fig. 3B) and transfected DNA was confirmed in electroporated primary cultured cells as preliminary data (Fig. S2B). Only lipofection with ScreenFectA showed a significantly higher ratio of EGF‐positive cells (13.2%) than controls (Fig. 3C). A genetic transfection was performed with scallop gonadal cells with active promoters (*PyActb*, *PyGpx*, *PyHsp70,* and *PyeIF3)* and transfection by lipofection (ScreenFectA).

### The tissue explant method allowed primary cultures of various cell types from various scallop organs

**Fig. 4:**
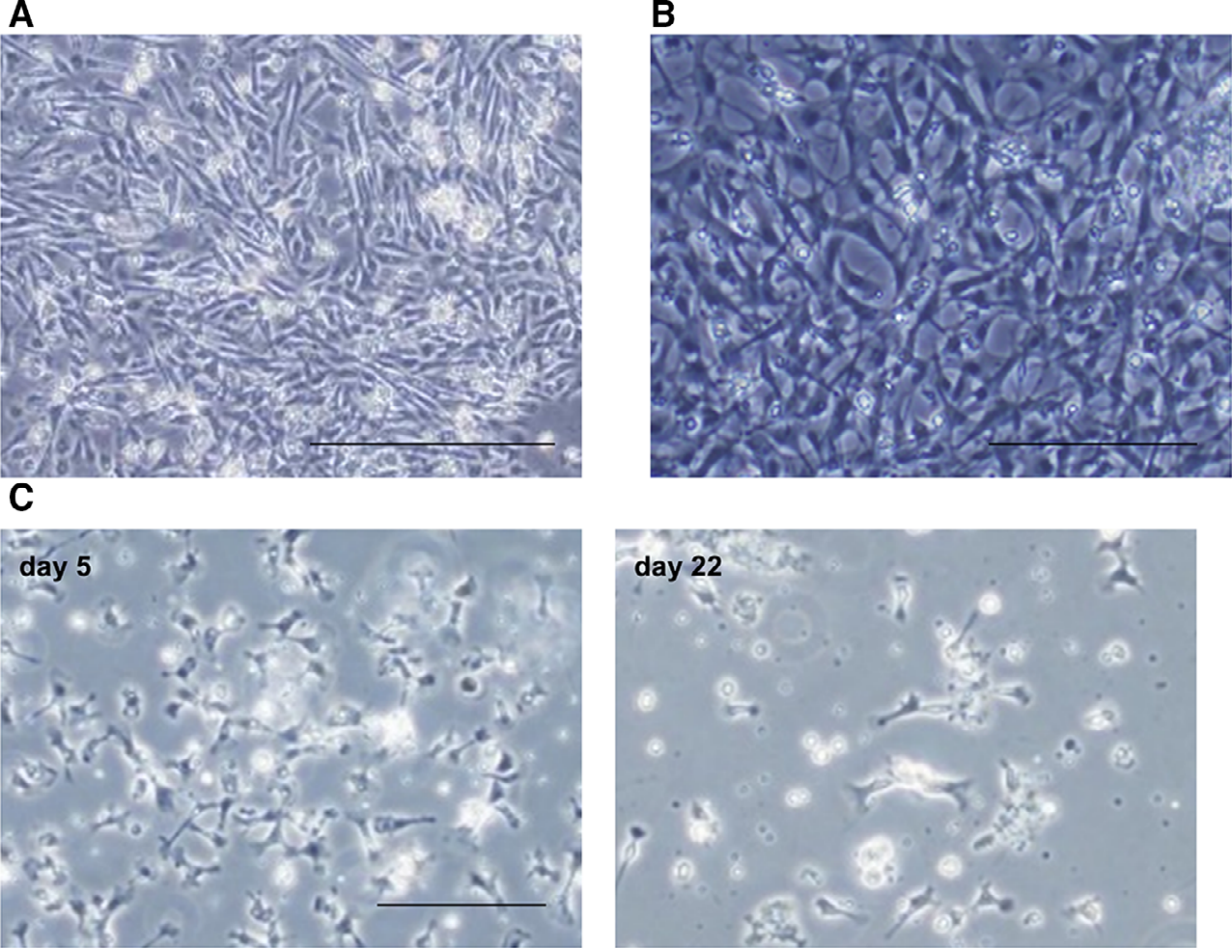
The tissue explant method allowed primary cultures of various cell types from various scallop organs. (A) Phase‐contrast images of hepatopancreatic cells at 3 days. Scale bar: 200 μm. (B) Phase‐contrast images of cultured adductor muscle cells. Scale bar: 100 μm. (C) Phase‐contrast images of cultured male gonadal cells at 5 and 22 days. Scale bar: 100 μm.

Because a cell dissociation method has not been developed for hepatopancreas, adductor muscle, or male gonad‐derived cells, the tissue explant method was adapted. A scallop hepatopancreas consists of many pouches and ducts. It contains the digestive tract and connects the mouth to the stomach (Fig. S3A). Blind pouches composed of some types of cells, secretory‐like cells that contain granules, basophils, which stained with hematoxylin, and adipocytes, which stained with oil red O (data not shown; Fig. S3A). In cell culture, hepatopancreatic cells adhered to gelatin‐coated dishes in M199 using the tissue explant method. However, cultured cells from hepatopancreas exfoliated a few days after removal of tissue from the dishes. Fibroblast‐like cells without filopodia were detected (Fig. 4A). Hepatopancreas‐derived cells were maintained 3 days after tissues were removed. The tissue‐specific markers, *PyPress* and *PyLec*, were isolated from all parts of the hepatopancreas (Fig. S3B). *PyPrss* encodes trypsinogen, the precursor of trypsin [18]. *PyLec* encodes a C‐type lectin domain family protein, which functions in the immune system in *Chlamys farreri* [19].

Adductor muscle cell culture was also attempted. Adductor muscle is composed of many polynuclear fiber bundles (Fig. S3C). In cell culture, mainly fibroblast‐like cells with MF were detected in gelatin‐coated dishes (Fig. 4B). The tissue‐specific marker, *PyMstn*, was also isolated. *PyMstn* encodes myostatin, which is highly expressed in skeletal muscle and which negatively regulates muscle proliferation in mammals (Fig. S3D) [20]. Primarily male gonadal cells with some filipodia were cultured (Fig. 4C). Male gonadal cells were maintained for 22 days (Fig. 4C).

## Discussion

We developed a primary culture system for female scallop gonadal cells in which cultured cells could be maintained for 1 month and had potential for proliferation. In addition, primary culture was also developed for hepatopancreas, adductor muscle, and male gonads. Furthermore, a transfection system was established for female gonadal cells with scallop‐derived promoters and lipofection reagent.

We found that modifying culture conditions to more closely mimic natural conditions improved scallop cell culture. Temperature and osmotic pressure have a significant influence on cell viability. Since scallop organs are exposed to the outside environment, it is reasonable to expect that they might be strongly affected by it [9]. In fact, temperature and salinity affect scallop survival *in vivo* [21].

We further succeeded in culturing scallop hepatopancreas, adductor muscle, and male gonad‐derived cells using the tissue explant method. Just as female gonadal cells tended to adhere around the cell mass, the tissue explant method enables other scallop cells to adhere. Cell masses or tissue fragments are advantageous in maintaining cell viability. One possible explanation is that tissue fragments may secrete specific nutrients or tissue factors for scallop cells. We found that AME enhanced cell viability. Furthermore, the tissue explant method seemed to be advantageous to reduce damage from cell dispersion and to expand the surface of extracellular matrix, which is important in primary culture for adherent cells [22]. In hepatopancreas‐derived cell culture, cells exfoliated a few days after removal of tissue from the dishes. This exfoliation may result from digestive enzymes secreted by the cells themselves, such as the trypsin precursor, *PyPress* [18].

The morphology of cultured scallop cells varied among organs. In both types of gonadal cells, most cultured cells were fibroblast‐like, consistent with previous findings [9]. In this study, round adhered cells and spread adhered cells were also observed in female gonadal cell culture. The spread adhered cells may be immune cells, since they had morphology similar to confluent granulocytes in *C. gigas* cultured heart cells[9]. Adductor muscular cells have MF. This feature is similar to myocytes, precursors of mature muscle cells in mice [23]. However, further examination is needed to identify phenotypes of cultured scallop cells by verifying gene expression.

Furthermore, we attempted transfection for female scallop gonadal cells, the first report for *P. yessoensis*. In fact, there are few reports of transfection in mollusks. Previous reports attempted transfection from larvae using various methods such as heat‐shock, electroporation, and pantropic retroviral vectors. Far fewer reports have been published regarding cultured cells, and transfection efficiency in these studies was low (0.5%) [24]. Species‐specific promoters enhance expression efficiency [25]; therefore, constructs of such promoters improved GFP expression in female gonadal cells. However, there is considerable room to improve the system because the EGFP expression level was low and varied among scallops. These primary cultures and this transfection technique will promote better understanding of scallop biochemical mechanisms and cellular structure, as well as providing clues for establishment of immortalized molluscan cell lines.

## Conflict of interest

There are no conflicts of interest to declare.

## Author contributions

YT and TN conceived and supervised the study; YT, TN, MS, and TO designed experiments; MS, TO, KU, and YI performed experiments; MS, TO, KU, YI, and TN analyzed data; MS and TN wrote the manuscript; TO, KU, YI, and YT made manuscript revisions.

## Supporting information


**Fig S1.** Screening of dish coating methods and detection system for cell number for primary culture of female scallop gonadal cells.
**Fig S2.** A schematic image and detection of transfected DNA for transfection methods of scallop gonadal cells with isolation of *P*. *yessoensis* promoters.
**Fig S3.** Histology and expression of the tissue‐specific genes of the scallop hepatopancreas and adductor muscle.

**Table S1.** Antibiotic components.
**Table S2.** Supplements for fundamental medium.
**Table S3.** Additional supplements.
**Table S4.**Additional supplements for UML‐15.
**Table S5.**Fundamental culture conditions for cells derived from hepatopancreas, adductor muscle, and male gonads.
**Table S6.** Primer arrangements for genomic PCR.
**Table S7.** Primer arrangements for RT‐PCR.

## Data Availability

The data in the present study are available from the corresponding author upon request.
