# Supplementary material for: Cell culture and genetic transfection methods for the Japanese scallop, Patinopecten yessoensis
Source: FEBS Open Bio. 2021 Jul 16;11(8):2282–91. doi: 10.1002/2211-5463.13237 (PMC8329786; doi:10.1002/2211-5463.13237)
Supplement: Supplementary file 1 — Fig S1. Screening of dish coating methods and detection system for cell number for primary culture of female scallop gonadal cells. Fig S2. A schematic image and detection of transfected DNA for transfection methods of scallop gonadal cells with isolation of P. yessoensis promoters. Fig S3. Histology and expression of the tissue‐specific genes of the scallop hepatopancreas and adductor muscle. [file FEB4-11-2282-s002.docx]

**Supplement figure 1. Screening of dish coating methods and detection system for cell number for primary culture of female scallop gonadal cells.**

**
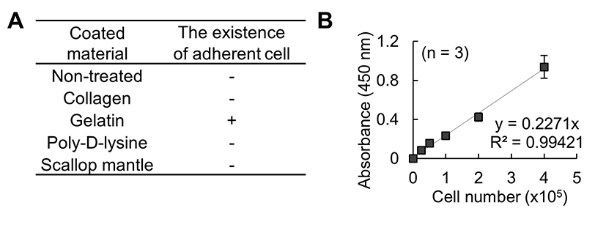
**

(A) Observation of adherent cells on each type of coating dish. (B) Correlation between CCK-8 absorbance of cultured cells and seeded numbers of cells before culture. Error bars: standard error, n=3.

**Supplemental figure 2.** **A schematic image and detection of transfected DNA for transfection methods of scallop gonadal cells with isolation of** **P. yessoensis promoters.**


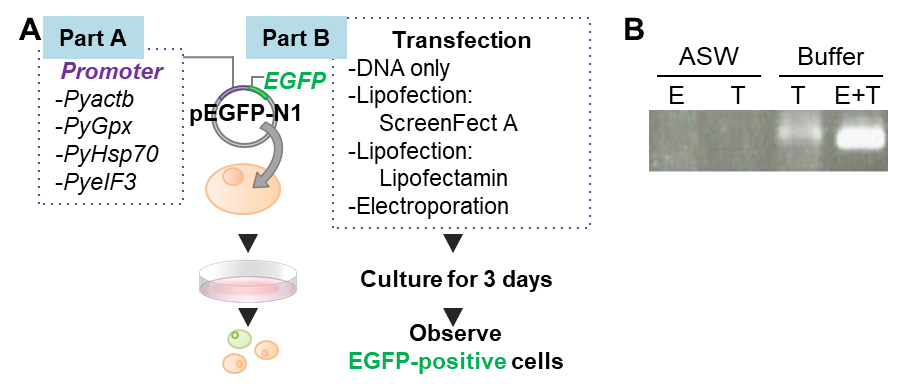


(A) A schematic image for transfection methods. (B) Detection of transfected DNAs with the primers for EGFP and electroporation in primary cultured scallop female gonadal cells (n=1). ASW: artificial sea water. Buffer: electroporation buffer. E: electrical pulse [Voltage (V):100, Pulse length (ms): 0.05, Number of pulses: 3, Pulse interval (sec): 0.1]. T: transfer pulse [Voltage (V): 25, Pulse length(ms): 50, Number of pulses: 10, Pulse interval (sec): 0.1].

**Supplemental figure 3.** **Histology and expression of the tissue-specific genes of the scallop hepatopancreas and adductor muscle.**

**
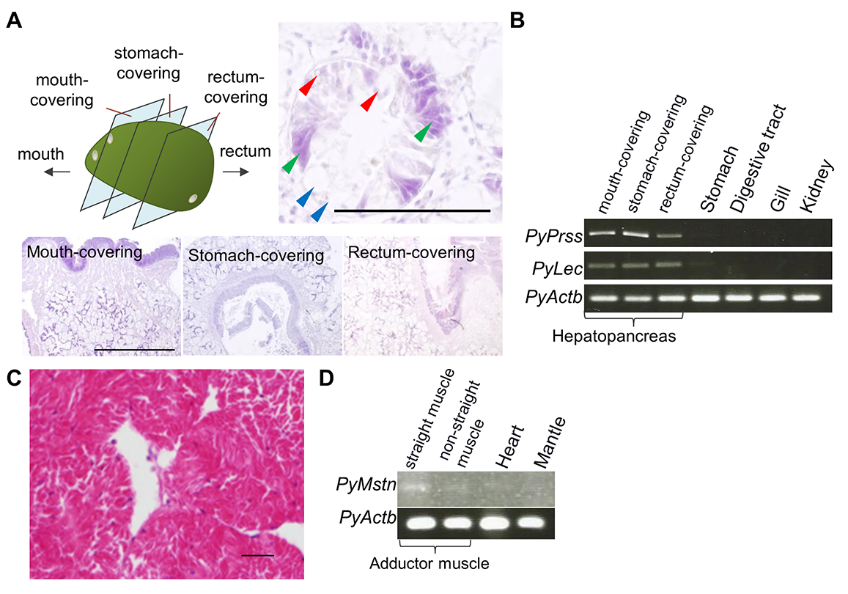
**

(A) Cross-sections and hepatopancreatic cell types at different positions. Red arrowheads; secretory-like cells, green arrowheads; basophil cells, blue arrowheads; fat-like cells. Scale bar: 2 mm in overview of the tissues and 200 μm in the ducts. (B) Gene expression of *PyPrss*, *PyLec*, and *PyActb* (internal control) in hepatopancreas and other organs. (C) A cross-section of adductor muscle. Scale bar 250 μm. (D)Expression of *PyMstn* and *PyActb* (internal control) in adductor muscle and other organs.
