# Supplementary material for: Cell culture and genetic transfection methods for the Japanese scallop, Patinopecten yessoensis
Source: FEBS Open Bio. 2021 Jul 16;11(8):2282–91. doi: 10.1002/2211-5463.13237 (PMC8329786; doi:10.1002/2211-5463.13237)
Supplement: Supplementary file 2 — Table S1. Antibiotic components. Table S2. Supplements for fundamental medium. Table S3. Additional supplements. Table S4.Additional supplements for UML‐15. Table S5.Fundamental culture conditions for cells derived from hepatopancreas, adductor muscle, and male gonads. Table S6. Primer arrangements for genomic PCR. Table S7. Primer arrangements for RT‐PCR. [file FEB4-11-2282-s001.docx]

| Antibiotics | Concentration (mg/mL) | Supplier |
| --- | --- | --- |
| Penicillin | 10 | Sigma, St Louis, MO, USA |
| Streptomycin | 10 | Sigma |
| Kanamycin | 10 | Fujifilm Wako, Osaka, Japan |
| Gentamicin | 12.5 | Nacalai Tesque, Kyoto, Japan |
| Tetracycline | 0.5 | Sigma |

Supplemental Table 1: Antibiotic components

Supplemental Table 2: Supplements for fundamental medium

| Supplements | Final concentration | Supplier |
| --- | --- | --- |
| GlutaMax | 1% | Life Technologies, Grand Island, NY, USA |
| pH 5.2 or 7.2 HEPES buffer | 100 μM |  |
| Reef Energy A | 0.5% | Red Sea, Israel |
| Reef Energy B | 0.5% | Red Sea |

Supplemental Table 3: Additional supplements

| Additional supplements | Final concentration | Supplier |
| --- | --- | --- |
| Adductor muscle extract | 1% |  |
| LiCl solution | each in below |  |
| LiCl (for activate Wnt signaling [1]) | 100 μM | Fujifilm Wako, Osaka, Japan |
| Bovine serum albumin (BSA)  (directly dissolved as final concentration) | 15 mg/mL | Iwai, Tokyo, Japan |
| Phosphoethanolamine (PE)  (maintenance of cell membrane [2]) | 100 μM | Sigma, St Louis, MO, USA |
| Tick-1 (referred as ML-15B in [3]) containing factors below | 1% |  |
| 3.3×10^-5^ M glutathione |  | Fujifilm Wako |
| 3.4×10^-3^ M sodium glutamate acid |  | Sigma |
| 12.4×10^-3^ M glucose |  | Sigma |
| 7.5×10^-5^ M ascorbic acid |  | Sigma |
| 2.6×10^-3^ M proline |  | Sigma |
| 1.0×10^-6^ M folic acid |  | Fujifilm Wako |
| 4.1×10^-7^ M biotin |  | Sigma |
| Tick-2 (modified Tick-1 only at concentration of folic acid and biotin) | 1% |  |
| 1.0×10^-3^ M folic acid |  | Fujifilm Wako |
| 4.1×10^-4^ M biotin |  | Sigma |
| Bovine lipoprotein-cholesterol  (BLC; referred as [4]) | 0.1% | MP Biomedicals, Irvine, CA, USA |
| Tryptose phosphate broth (TP; referred as [4]) | 5% | Dickinson and Company, NJ, USA |

1 Clevers H & Nusse R (2012) Wnt/β-catenin signaling and disease. *Cell* **149**, 1192–1205.

2 Kano‐Sueoka T, King DM, Fisk HA & Klug SJ (1990) Binding of epidermal growth factor to its receptor is affected by membrane phospholipid environment. *J Cell Physiol* **145**, 543–548.

3 Munderloh UG & Kurtti TJ (1989) Formulation of medium for tick cell culture. *Exp Appl Acarol* **7**, 219–229.

4 Goblirsch MJ, Spivak MS & Kurtti TJ (2013) A Cell Line Resource Derived from Honey Bee (*Apis mellifera*) Embryonic Tissues. *PLoS One* **8**, 1–13.

Supplemental Table 4: Additional supplements for UML-15

| Additional supplements for UML-15 | Final concentration |
| --- | --- |
| Solution 1: diluted in water | 1% |
| 3.3×10^-5^ M glutathione |  |
| 3.4×10^-3^ M sodium glutamate acid |  |
| 12.4×10^-3^ M glucose |  |
| 7.5×10^-5^ M ascorbic acid |  |
| 2.6×10^-3^ M proline |  |
| 10 mM LiCl |  |
| Solution 2: diluted in 1 N NaOH | 1% |
| 1.0×10^-3^ M folic acid |  |
| 4.1×10^-4^ M biotin |  |
| Solution 3 |  |
| 100 mM phosphoethanolamine | 0.1% |
| BSA | 15 mg/mL |

Supplemental Table 5: Fundamental culture conditions for cells derived from hepatopancreas, adductor muscle, and male gonads

|  | Hepatopancreas | Adductor muscle | Male gonad |
| --- | --- | --- | --- |
| Coat substance | gelatin | gelatin | gelatin |
| Medium | M199 | MNCTC-135 | UML-15 |
| pH | 5.8 | 5.2 | 5.2 |
| Osmolality | 860 | 400 | 400 |

Supplemental Table 6: Primer arrangements for genomic PCR

5 Wang S, Zhang J, Jiao W, Li J, Xun X, Sun Y, Guo X, Huan P, Dong B, Zhang L, Hu X, Sun X, Wang J, Zhao C, Wang Y, Wang D, Huang X, Wang R, Lv J, Li Y, Zhang Z, Liu B, Lu W, Hui Y, Liang J, Zhou Z, Hou R, Li X, Liu Y, Li H, Ning X, Lin Y, Zhao L, Xing Q, Dou J, Li Y, Mao J, Guo H, Dou H, Li T, Mu C, Jiang W, Fu Q, Fu X, Miao Y, Liu J, Yu Q, Li R, Liao H, Li X, Kong Y, Jiang Z, Chourrout D, Li R & Bao Z (2017) Scallop genome provides insights into evolution of bilaterian karyotype and development. *Nat Ecol Evol* **1**.

| Primer arrangements 5’ to 3’ | | | Annealing temp.  (℃) | Cycles |
| --- | --- | --- | --- | --- |
| *PyActb*  (reffered as [5]) | Fw. | TGGGGCGATGATTCAACAGCAAGAT | 60 | 35 |
|  | Rv. | ACAGCTTTCCGAGAACTCCACGAA |  |  |
| *PyeIF3*  (reffered as [5]) | Fw. | ACAGAATTGGTGAGCTTGCCAGACC | 60 | 35 |
|  | Rv. | CAGGTACACCCGTACGCATACAAACA |  |  |
| *PyGpx*  (reffered as [5]) | Fw. | TCACTTGAAGACGCTTGTTGTCGGT | 60 | 36 |
|  | Rv. | CAGGCGTGACATGAGGTATTTTCGT |  |  |
| *PyHsp70*  (reffered as [5]) | Fw. | ACGAAGCTTTTGTTCTTGCAGGTCA | 60 | 35 |
|  | Rv. | CCCGTATTTATGCGCACCAGAGGAT |  |  |

Supplemental Table 7 Primer arrangements for RT-PCR

| Primer arrangements 5’ to 3’ | | | Annealing temp. | Cycles |
| --- | --- | --- | --- | --- |
|  |  |  | (℃) |  |
| *PyActb*  (referred as PubMed accession No. DQ787858) | Fw. | TTCGAGAGCGAGATGAGCA | 58 | 32 |
|  | Rv. | AGAGAGATTCGGGACACCTG |  |  |
| *PyPrss*  (referred as [6]) | Fw. | CCTCAGGCACCCATCTGTTT | 58 | 32 |
|  | Rv. | GATTACCCAGCGACCAGACA |  |  |
| *PyLec*  (referred as [6]) | Fw. | ACGGTGGATGTCCATTTGGT | 58 | 27 |
|  | Rv. | AGCATCAATGGGCCTGTCAA |  |  |
| *PyMstn*  (referred as [6]) | Fw. | TACTACTGCGCTGGGG | 58 | 40 |
|  | Rv. | TCGAGACCAAAAAGGCGAAGG |  |  |

6 Meng X lin, Liu M, Jiang K yong, Wang B jie, Tian X, Sun S juan, Luo Z yong, Qiu C wen & Wang L (2013) De Novo Characterization of Japanese Scallop *Mizuhopecten yessoensis* Transcriptome and Analysis of Its Gene Expression following Cadmium Exposure. *PLoS One* **8**.
